# Supplementary material for: mbtransfer: Microbiome intervention analysis using transfer functions and mirror statistics
Source: PLoS Comput Biol. 2024 Jun 14;20(6):e1012196. doi: 10.1371/journal.pcbi.1012196 (PMC11210883; doi:10.1371/journal.pcbi.1012196)
Supplement: S1 Text — Discussion of simulation setup, including details of the data generation mechanism and hyperparameter grid used to create contrasting datasets. It also provides mathematical details of the evaluation criteria and an overview of the MDSINE2 and fido algorithms. (PDF) [file pcbi.1012196.s001.pdf]

# Supplementary Information for "mbtransfer: Microbiome intervention analysis using transfer functions and mirror statistics"

## A Simulation details

### A.1 Model Parameters

The $J$ dispersion parameters are simulated according to $\varphi_j \sim \text{Unif}[1, 5]$. This ensures that the simulated counts are overdispersed while lying in a realistic range for microbiome data. The parameters $B_q, C_q$, and $A_p$ are simulated as follows. The first $J_1$ taxa are assumed to have true intervention effects and the remaining $J_0 = J - J_1$ rows of $B_q$ and $C_q$ are set to 0. Among the nonnull taxa $j \in J_1$, we draw $B_{q,jd} \sim \text{Unif}([-2b, -b] \cup [b, 2b])$ where $b$ encodes the signal strength. Using two intervals ensures that nonnull effects are bounded away from 0. Entries $C_{q,jd}$ are drawn similarly, except entire rows $C_{q,j\cdot}$ are set to 0 with an additional probability $p_c$. Such rows represent taxa with real intervention effects but no interaction with host characteristics, represented by $z^{(i)} \sim \mathcal{N}\left(0, \sigma_z^2\right)$. Finally, we simulate $A \in \mathbb{R}^{J \times J}$ as a sparsified version of a random, low-rank matrix. This ensures that interaction effects are related across sub-communities and that most pairs taxa remain unaffected by one another. Specifically, we first set $\tilde{A}^{(0)} \sim QQ^T$ where $Q \in \mathbb{R}^{J \times K}$ has entries drawn independently from $\mathcal{N}\left(0, \sigma_A^2\right)$. Entries of $\tilde{A}^{(0)}$ are randomly set to 0 with probability $p_A$, yielding $\tilde{A}^{(1)}$, and the result is normalized: $A = \frac{\tilde{A}^{(1)}}{\|\tilde{A}^{(1)}\|_2}$.

### A.2 Data Parameters

We vary the following data parameters:

1. The number of taxa $\in \{100, 200, 400\}$.
2. The fraction of null taxa $\pi_0 \in \{0.1, 0.2, 0.4\}$. Given $\pi_0$, we set $J_0 = \lfloor \pi_0 J \rfloor$.
3. The signal strength $b \in \{0.25, 0.5, 1\}$.
4. The phylogenetic correlation $\alpha \in \{0.1, 10\}$
5. The sampling depth heterogeneity $\lambda \in \{0.1, 10\}$

Across all runs, we simulate 50 subjects with 30 timepoints each. We fix $p_c = 0.2$ and $p_A = 0.4$. Considering all combinations of these parameters yields 108 simulated datasets. These can be downloaded from https://go.wisc.edu/8ey754, and intermediate simulation outputs are available at https://go.wisc.edu/3gc982.

We simulate random, one-dimensional interventions $w_t^{(i)} \in \{0, 1\}$ by first randomly sampling a starting point $t^{\text{start}} \sim \text{Unif}\left[\frac{T}{3}, \ldots, \frac{2T}{3}\right]$. The intervention length is drawn from $\ell \in \text{Unif}[L, 2L]$. If $t^{\text{start}} + \ell > T$, we truncate the intervention series at $T$. This creates heterogeneity in both the start time and duration of the interventions, a common property across many microbiome community studies.

### A.3 Evaluation Strategy

To assess forecasting performance, we first divide the 50 simulated subjects into $K = 4$ cross-validation folds. For each $k$, models are trained with the $\mathbf{Y}_{t-1}^{(i)}, \mathbf{W}_t^{(i)}$ from all subjects except those in the holdout fold. On holdout folds, we reveal all timepoints up to the first intervention $t^*$ in the currently held-out subject. The

trained models then forecast the community profiles up to a time horizon of $H = 5$. We provide access to intermediate interventions $\mathbf{W}_{t+h}^{(i)}$, but not community compositions $\mathbf{y}_{t+h}^{(i)}$ for $h > t^*$. For each $k$, we compute the mean absolute error across lags and holdout subjects:

$$MAE_k = \frac{1}{JH} \frac{1}{|\mathcal{D}_{-k}|} \sum_{h=1}^{H} \sum_{d_i \in \mathcal{D}_{-k}} \left\| \mathbf{y}_{t^*+h}^{(i)} - \hat{\mathbf{y}}_{t^*+h}^{(i)} \right\|_1 .$$

Next, we consider evaluation of inferential performance. To compute the false discovery proportion and power associated with instantaneous effects (lag $h = 0$), we use

$$\text{FDP}(0) = \frac{\left|J_0 \cap \hat{J}(0)\right|}{\left|\hat{J}(0)\right|} \qquad \text{Power}(0) = \frac{\left|J_1 \cap \hat{J}(0)\right|}{|J_1|},$$

where $\hat{J}(0)$ are the taxa flagged as having immediate intervention effects and $J_1(0)$ are the rows of $B_0$ with at least one nonnull effect $\{j : B_{0,jd} \neq 0 \text{ for some } d\}$. For delayed effects, we must account both for taxa with nonzero entries of $B_q$ and also those taxa that are indirectly shifted by autoregressive links $A_p$ with taxa that are affected by the intervention. To this end, we recursively define:

$$J_1(h) = \left\{ j : \text{row } j \text{ of } \prod_{p=1}^{h} A_p \mathbf{1}_{J_1(h-p)} \text{ has at least one nonzero element} \right\} \bigcup \left\{ j : B_{h,jd} \neq 0 \text{ for some } d \right\},$$

where $\mathbf{1}_{J_1(h-p)} \in \{0,1\}^J$ is an indicator over taxa that are nonnull at lag $h - p$. The selected taxa at delay $h$ are denoted $\hat{J}(h)$, and they can be compared with $J_1(h)$ to define $\text{FDP}(h)$ and $\text{Power}(h)$.

# B Reproducibility

## Simulation experiments

- A Dockerfile that installs software used in the experiments is available at https://go.wisc.edu/eovk4b. The image can be pulled from DockerHub using `docker pull krisrs1128/mi:20230506`.
- Simulation inputs have been saved at https://go.wisc.edu/8ey754. They were generated using https://go.wisc.edu/37y6n This script also includes source code for Fig 3.
- Each forecasting and inference simulation run corresponds to one `run_id` of these Rmarkdown notebooks: I, II. Simulation outputs have been saved at https://go.wisc.edu/3gc982.
- Figs 4, 6, and Figs E - F in S2 Text were generated using this script https://go.wisc.edu/87876s applied to the previous outputs. Fig 5 was generated using https://go.wisc.edu/l1n79m.

# Case studies

- The case studies appear as vignettes in our accompanying R package (https://go.wisc.edu/crj6k6, I, II). They can also be rerun without installing the package by visiting this binder notebook: https://go.wisc.edu/emxv33.
- Processed versions of the data used in all case studies can be found on figshare: https://go.wisc.edu/7ig8q8, https://go.wisc.edu/83l84r. The data were processed according to this script https://go.wisc.edu/37x8hh.

# C Summary of MDSINE2 and FIDO

This section briefly describes the MDSINE2 [1, 2] and fido [3] methods that were used in the simulation study.

## MDSINE2

MDSINE2 is a recently proposed Bayesian model of microbiome dynamics. It adapts the generalized Lotka-Volterra dynamics in the following ways,

1. Taxa are assigned to clusters. This effectively reduces the dimensionality of the gLV's autoregressive dynamics – taxa influence one another's growth rates via their cluster membership. Moreover, perturbation effects are constrained to be identical across all taxa within the same cluster.

2. The approach is probabilistic and models each sample's total count and relative abundance structure. This contrasts with alternative gLV estimators, which often proceed by initially transforming abundance and applying regularized least squares.

The model's generative mechanism is as follows. If we assume uniform sampling over time, then the model generates latent taxonomic abundances according to

$$\log x_j^{(i)}(t+1) \,|\mu_j^{(i)}(t) \sim \mathcal{N}\left(\log \mu_{s,k}(t+1), \sigma^2\right)$$

where $i, j$ and $t$ index subjects, taxa, and time. The mean vector $\mu_j^{(i)}$ is a deterministic, gLV-like function of random clustering and growth parameters,

$$\log \mu_j^{(i)}(t+1) := \log x_j^{(i)}(t) + a_{1,j}\left[1 + \sum_{p=1}^{P} \gamma_{c_j} \mathbf{z}_{c_j,p}^{(\gamma)} \mathbf{1}\left\{(i,t) \in \texttt{Perturbation } p\right\}\right] - a_{2,j} x_j^{(i)}(t) + \sum_{j':c_{j'} \neq c_j} b_{c_j c_{j'}} \mathbf{z}_{c_j c_{j'}}^{(b)} x_{j'}^{(i)}(t)$$

The terms $a_{1,j}$ and $a_{2,j}$ are the growth and decay rates for taxon $j$, as in the standard gLV. $c_j$ is the cluster index of taxon $j$. The summation of perturbations $p$ describes the influence of different perturbations on taxon $j$'s abundance. $\gamma_{c_j}$ and $\mathbf{z}_{c_j,p}^{(\gamma)}$ parameterized the strength and presence of a perturbation $p$ effect on cluster $c_j$ – note that the perturbation influences are shared across all members of the same cluster. The final summation describes autoregressive dynamics between pairs of taxa $j, j'$. The autoregressive coefficients are shared between all pairs of taxa with the same cluster assignments $c_j, c_{j'}$. In this way, the autoregressive dynamics operate at the cluster level.

These latent taxonomic abundances $x_j^{(i)}(t)$ are transformed into the observed relative $y_j^{(i)}(t)$ and total $r_i(t)$ abundances for taxon $j$ in sample $t$ of subject $i$ using a negative binomial measurement model,

$$y_j^{(i)}(t) \,|\left(x_j^{(i)}(t)\right)_{j=1}^{J} \sim \text{NB}\left(\frac{r_i(t)\, x_j^{(i)}(t)}{\sum_{j'} x_{j'}^{(i)}(t)}, d_1 + d_0\left(\frac{x_j^{(i)}(t)}{\sum_{j'} x_{j'}^{(i)}(t)}\right)^{-1}\right)$$

where $d_0$ and $d_1$ are hyperparameters set in advance to account for dispersion in the observed samples.

Finally, priors are placed on the parameters $a_{1,j}, a_{2,j}, c_j, \gamma_l, \mathbf{z}_l^{(b)}, \mathbf{z}_l^{(\gamma)}$, and $b_{ll'}$. A stick-breaking process prior is placed on $c_j$, allowing for the number of clusters to adapt to the evidence in the data. Inference is performed through MCMC, cycling over these parameters and those used within hyperpriors.

### fido

fido combines a multinomial logistic-normal model, matrix-normal process, and Bayesian inference to model the effect of covariates on taxa abundance.

- The multinomial proportions are transformed into real space. Then, the mean of the transformed data is assumed to be latent matrix-normal processes. This approach allows for the modeling of latent factors that capture the shared information across the taxa.
- The Bayesian framework starts with specifying priors for taxa covariance. Then, during each iteration of the collapse-uncollapse sampler, the `fido` updates the latent factors that capture the shared information across the taxa using the latent - T process (LTP). In addition, the regression coefficients are updated to relate the latent factors and covariates, including perturbations.

Let's assume there are $J$ taxa, $Q$ covariates, and $N$ total samples. The model generative process for $k$-th taxa abundance at time $t$ in subject $i$, $y_j^{(i)}(t)$ is as follows.

1. The prior distribution of the covariance between additive log-ratio transformed (ALR) taxa is inverse Wishart distribution, $\Sigma_{J-1\times J-1} \sim W^{-1}(\Xi, \upsilon)$ with a scale matrix $\Xi_{J-1\times J-1}$ and degrees of freedom $\upsilon$.
2. Then, the smooth mean function $\Lambda[X] \sim \mathrm{GP}(\Theta[X], \Sigma, \Gamma[X])$ relates the covariates $X_{Q\times N}$ to ALR-transformed $\eta$ with the mean function $\Theta[X]$, row (taxa) covariance $\Sigma$, and column (samples) covariance $\Gamma[X]$.
   - Covariates $X$ includes time $t$.
   - $\Gamma[X]$ evaluates the kernel $K$ at timepoints $t$ and $t-1$ for a given subject $s$, $K(X_s(t), X_s(t-1))$ otherwise, it is zero.
3. Next, $\eta \sim \mathrm{N}(\Lambda[X], \Sigma, I_N)$ is a normal-matrix distribution, where $\pi = \phi^{-1}(\eta)$ and $\eta$ is a $(J-1)\times N$ real valued matrix.
4. For the subject $i$ at time $t$, we compute the inverse of ALR, $\pi^{(i)}(t)$ and generate $\mathbf{y}^{(i)}(t)$

$$\mathbf{y}^{(i)}(t)\,|\pi^{(i)}(t) \sim \mathrm{Multinomial}\left(n^{(i)}(t), \pi^{(i)}(t)\right),$$

   where $n^{(i)}(t)$ is the library size.

# References


[1] Bucci V, Tzen B, Li N, Simmons M, Tanoue T, Bogart E, et al. MDSINE: Microbial Dynamical Systems INference Engine for microbiome time-series analyses. Genome Biology. 2016;17.

[2] Gibson TE, Kim Y, Acharya S, Kaplan DE, DiBenedetto N, Lavin R, et al. Intrinsic instability of the dysbiotic microbiome revealed through dynamical systems inference at scale. bioRxiv. 2021;.

[3] Silverman JD, Roche K, Holmes ZC, David LA, Mukherjee S. Bayesian Multinomial Logistic Normal Models through Marginally Latent Matrix-T Processes. Journal of Machine Learning Research. 2022;23. Available from: `https://www.jmlr.org/papers/v23/19-882.html`.
