# Supplementary material for: mbtransfer: Microbiome intervention analysis using transfer functions and mirror statistics
Source: PLoS Comput Biol. 2024 Jun 14;20(6):e1012196. doi: 10.1371/journal.pcbi.1012196 (PMC11210883; doi:10.1371/journal.pcbi.1012196)
Supplement: S2 Text — Numerical summaries and visualization of other settings in the simulation and case studies. (PDF) [file pcbi.1012196.s002.pdf]

Supplementary Tables and Figures for "mbtransfer: Microbiome intervention analysis using transfer functions and mirror statistics"

**lag** h	**Abundance Quantile**	**Correlation (within subject)**	**Correlation (new subjects)**
1	(3.65,9.44]	0.8960	0.5578
2	(3.65,9.44]	0.8331	0.5019
3	(3.65,9.44]	0.8208	0.4352
4	(3.65,9.44]	0.8087	0.4764
5	(3.65,9.44]	0.8249	0.4861
1	(1.95,3.65]	0.8673	0.5489
2	(1.95,3.65]	0.7968	0.4137
3	(1.95,3.65]	0.7358	0.2670
4	(1.95,3.65]	0.7322	0.3847
5	(1.95,3.65]	0.7637	0.3538
1	(0.391,1.95]	0.8556	0.4051
2	(0.391,1.95]	0.7922	0.4421
3	(0.391,1.95]	0.7484	0.1443
4	(0.391,1.95]	0.7311	0.1856
5	(0.391,1.95]	0.7328	0.2064
1	[0,0.391]	0.8444	0.3442
2	[0,0.391]	0.8263	0.3641
3	[0,0.391]	0.7294	0.1233
4	[0,0.391]	0.7630	0.2665
5	[0,0.391]	0.7540	0.2747

Table A: Pearson correlation between $\mathbf{y}_{t+h}^{(i)}$ and $\hat{\mathbf{y}}_{t+h}^{(i)}$ when using mbtransfer for the diet intervention study. Deterioration in correlation in new subjects reflects high intersubject heterogeneity in microbiome composition. Correlations are also generally stronger for the more abundant taxa.

lag h	**Abundance Quantile**	**Correlation (within subject)**	**Correlation (new subjects)**
1	(3.65,9.44]	0.8155	0.8102
2	(3.65,9.44]	0.7007	0.6393
3	(3.65,9.44]	0.4924	0.4711
4	(3.65,9.44]	0.3334	0.3210
5	(3.65,9.44]	0.2209	0.2439
1	(1.95,3.65]	0.7775	0.7737
2	(1.95,3.65]	0.6440	0.6132
3	(1.95,3.65]	0.4092	0.3746
4	(1.95,3.65]	0.2209	0.1885
5	(1.95,3.65]	0.1265	0.0945
1	(0.391,1.95]	0.7375	0.7380
2	(0.391,1.95]	0.6724	0.6730
3	(0.391,1.95]	0.4806	0.5233
4	(0.391,1.95]	0.2514	0.3436
5	(0.391,1.95]	0.1319	0.2644
1	[0,0.391]	0.7229	0.6518
2	[0,0.391]	0.7214	0.6498
3	[0,0.391]	0.5565	0.4618
4	[0,0.391]	0.4618	0.3407
5	[0,0.391]	0.4156	0.3113

Table B: Pearson correlation between $\mathbf{y}_{t+h}^{(i)}$ and $\hat{\mathbf{y}}_{t+h}^{(i)}$ when using MDSINE2 on the diet intervention study. Each row corresponds to one panel from H. Performance is comparable to mbtransfer for shorter lags but deteriorates on longer time horizons. Forecasts for abundant taxa and new subjects are better when using MDSINE compared to either mbtransfer or fido.

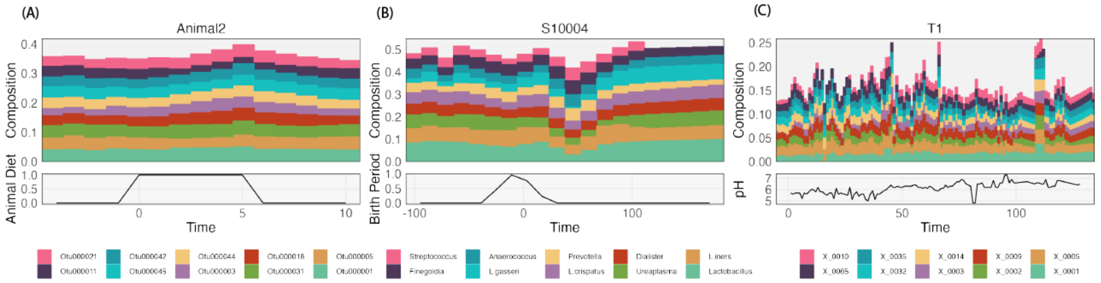

Fig A: Examples of microbial community shifts in response to environmental change. Part (A) describes the gut microbiome of a subject undergoing a diet intervention [2], (B) shows the remodeling of a mother's vaginal microbiome following birth [1], and (C) profiles an aquaculture tank microbiome together with environmental pH [3].

**lag** h	**Abundance Quantile**	**Correlation (within subject)**	**Correlation (new subjects)**
1	(3.65,9.44]	0.7433	0.7662
2	(3.65,9.44]	0.7537	0.7419
3	(3.65,9.44]	0.7220	0.7138
4	(3.65,9.44]	0.7000	0.6946
5	(3.65,9.44]	0.7227	0.7374
1	(1.95,3.65]	0.7473	0.7577
2	(1.95,3.65]	0.7500	0.7521
3	(1.95,3.65]	0.7066	0.7112
4	(1.95,3.65]	0.6960	0.7392
5	(1.95,3.65]	0.7474	0.7775
1	(0.391,1.95]	0.7583	0.7766
2	(0.391,1.95]	0.7416	0.7528
3	(0.391,1.95]	0.7240	0.7031
4	(0.391,1.95]	0.6897	0.6939
5	(0.391,1.95]	0.7234	0.7314
1	[0,0.391]	0.7716	0.7348
2	[0,0.391]	0.7964	0.7928
3	[0,0.391]	0.7549	0.7415
4	[0,0.391]	0.7800	0.7659
5	[0,0.391]	0.7753	0.7641

Table C: Pearson correlation between $\mathbf{y}_{t+h}^{(i)}$ and $\hat{\mathbf{y}}_{t+h}^{(i)}$ when using fido on the diet intervention study. Each row corresponds to one panel from S2 Fig I. Fido performs well in new subjects relative to mbtransfer and MDSINE2. Forecasting for previously observed subjects is somewhat lower for shorter time horizons and more abundant taxa.

**lag** h	**Abundance Quantile**	**Correlation (within subject)**	**Correlation (new subjects)**
0	(5.48,11.5]	0.7862	0.7378
10	(5.48,11.5]	0.7073	0.5924
20	(5.48,11.5]	0.5162	0.3225
30	(5.48,11.5]	0.5193	0.4404
40	(5.48,11.5]	0.5636	0.4751
0	(2.94,5.48]	0.6924	0.5983
10	(2.94,5.48]	0.5970	0.4768
20	(2.94,5.48]	0.5059	0.3734
30	(2.94,5.48]	0.4770	0.2725
40	(2.94,5.48]	0.5034	0.2934
0	(0.547,2.94]	0.7289	0.5654
10	(0.547,2.94]	0.6665	0.5067
20	(0.547,2.94]	0.6143	0.4095
30	(0.547,2.94]	0.5351	0.4235
40	(0.547,2.94]	0.5809	0.3861
0	[0,0.547]	0.8491	0.7763
10	[0,0.547]	0.7726	0.7020
20	[0,0.547]	0.6457	0.4694
30	[0,0.547]	0.5023	0.5132
40	[0,0.547]	0.5454	0.4553

Table D: Pearson correlation between $\mathbf{y}_{t+h}^{(i)}$ and $\hat{\mathbf{y}}_{t+h}^{(i)}$ when using mbtransfer on the postpartum study, quantifying the effects visible in Fig. J. Performance for abundant taxa at shorter time horizon is better than for rarer taxa and longer time horizons.

**lag** h	**Abundance Quantile**	**Correlation (within subject)**	**Correlation (new subjects)**
0	(5.48,11.5]	0.6994	0.1725
10	(5.48,11.5]	0.6199	0.1024
20	(5.48,11.5]	0.6616	0.0734
30	(5.48,11.5]	0.6896	-0.0307
40	(5.48,11.5]	0.6167	0.0290
0	(2.94,5.48]	0.6389	0.0505
10	(2.94,5.48]	0.5859	0.1823
20	(2.94,5.48]	0.6489	0.2136
30	(2.94,5.48]	0.6447	0.0599
40	(2.94,5.48]	0.6558	-0.0051
0	(0.547,2.94]	0.5801	0.2069
10	(0.547,2.94]	0.6159	-0.0169
20	(0.547,2.94]	0.6933	-0.046
30	(0.547,2.94]	0.6940	-0.0131
40	(0.547,2.94]	0.6884	-0.0015
0	[0,0.547]	0.7929	0.0880
10	[0,0.547]	0.7306	0.1841
20	[0,0.547]	0.7893	-0.0568
30	[0,0.547]	0.7073	0.0022
40	[0,0.547]	0.7194	0.0465

Table E: Pearson correlation between $\mathbf{y}_{t+h}^{(i)}$ and $\hat{\mathbf{y}}_{t+h}^{(i)}$ when using fido on the postpartum study. Each row corresponds to a panel from S2 Fig K. Missing values for predictions on new subjects have been imputed with 0. In contrast to the diet intervention, within subject performance is substantially higher than out-of-subject prediction.

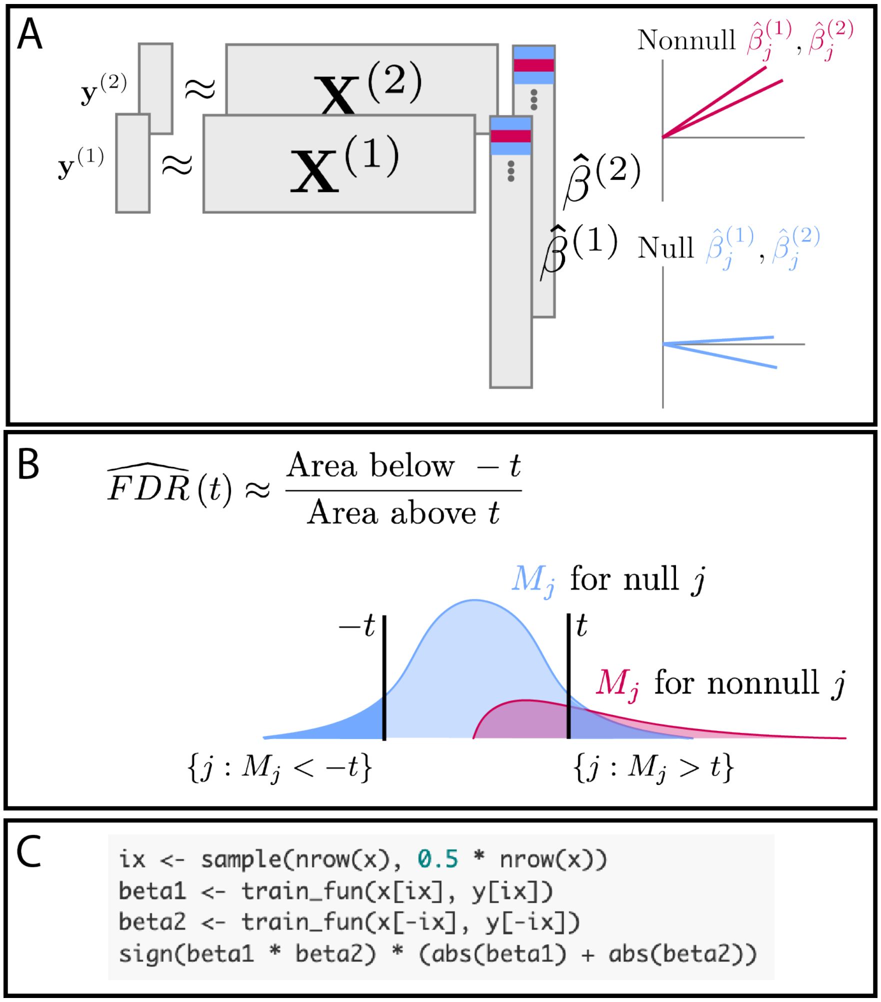

Fig B: Intuition behind the mirror statistics algorithm in a linear regression setting. (A) The mirror algorithm first randomly splits the full dataset into $\left(\mathbf{X}^{(1)}, \mathbf{y}^{(1)}\right)$ and $\left(\mathbf{X}^{(2)}, \mathbf{y}^{(2)}\right)$ For a coordinate β_j with a real effect, we expect the signs of the estimates $\hat{\beta}_j^{(1)}$ and $\hat{\beta}_j^{(2)}$ to agree. (B) Under the null, the critical assumption is that M_j is symmetric under the null. This makes it possible to estimate the FDR of a rule that selects effects when $M_j > t$ by referring to the area $M_j < -t$. The estimated false discovery rate is the number of hypotheses lying in the blue area on the left divided by the number of hypotheses in the mixed red and blue area on the right. This works because the area on the right refers to the total number of discoveries, while the blue area on the left gives an estimate for the blue area on the right, which is the actual false discoveries. (C) Pseudocode for calculation of the statistics M_j for one split of the data.

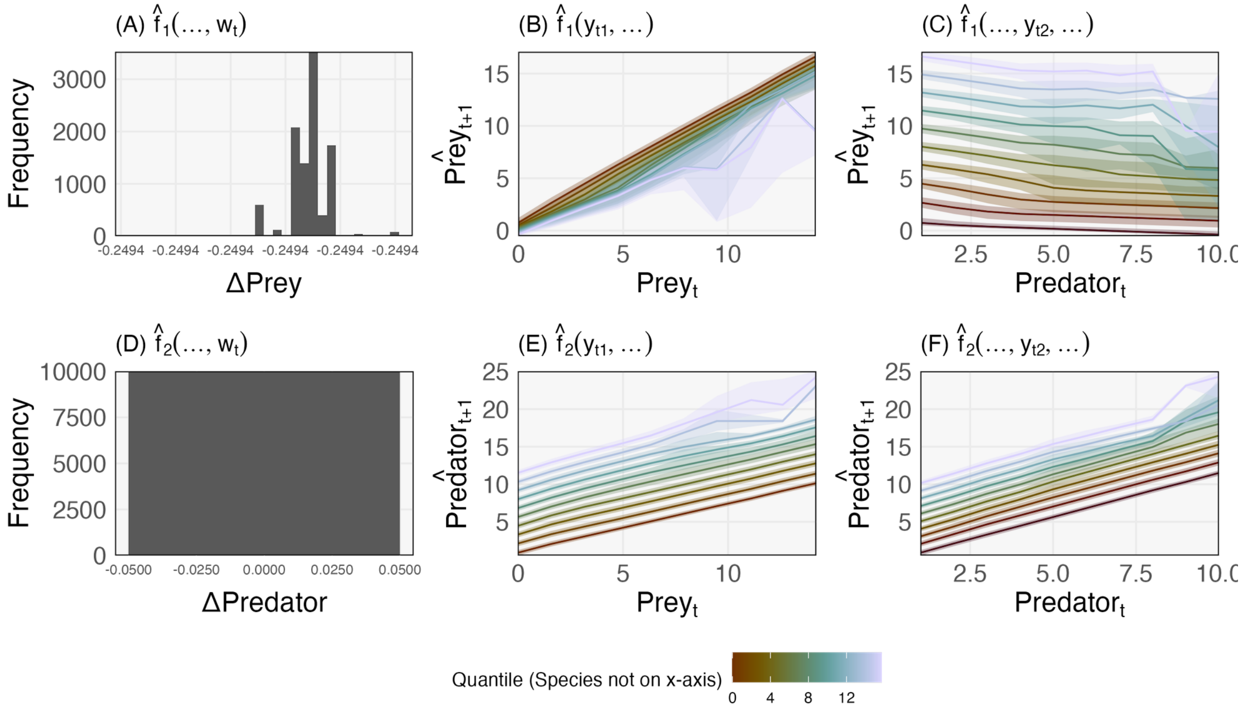

Fig C: Partial dependence profiles for the transfer function model applied to the predator-prey example of Fig 2. The top and bottom rows correspond to $\hat{f}_j$ with prey and predator as response, respectively. The model has learned that the perturbation w_1 results in an instantaneous decrease in prey (A) but not predator (B) populations. The directionality of all profiles reflects true predator-prey dynamics. Interactions are present, though generally weaker than the true simulation mechanism.

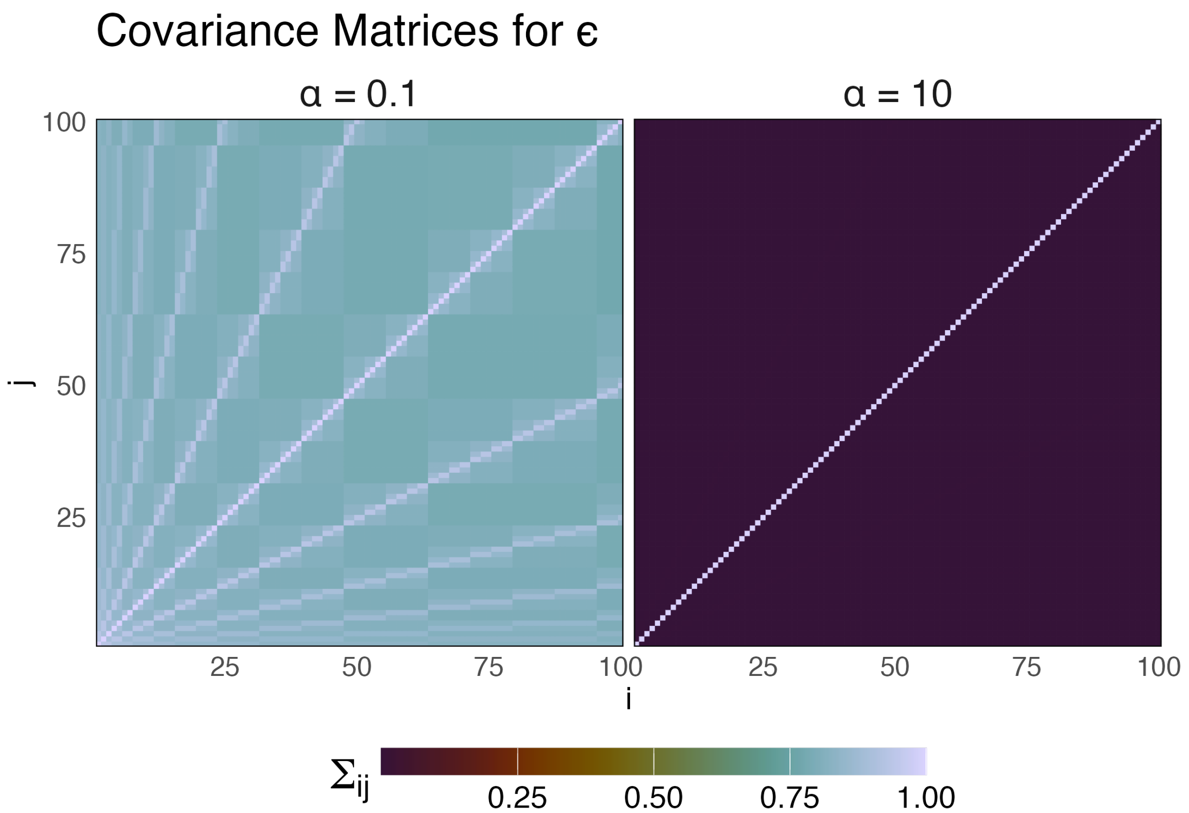

Fig D: The tree-induced covariance matrices for $\epsilon^{(i)}$ used in the simulation study. When $\alpha = 0.1$, errors across taxa are highly correlated, while for $\alpha = 10$, the errors are essentially independent.

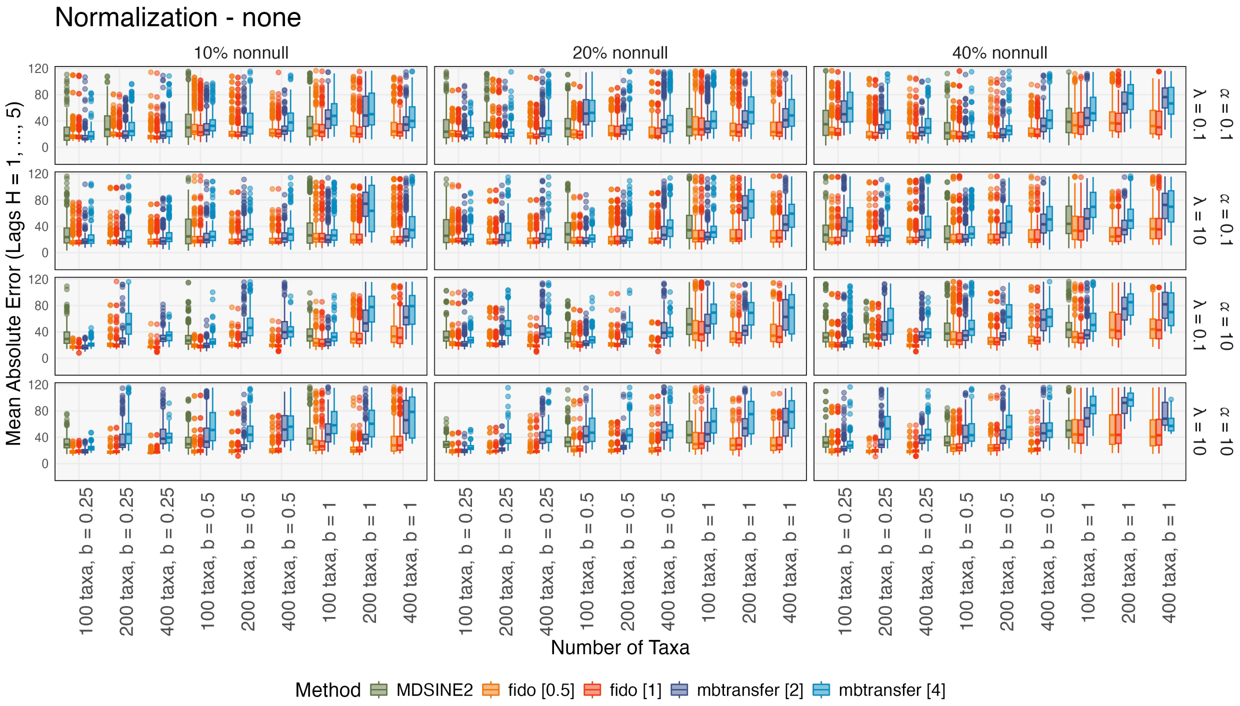

Fig E: The analog of Fig 4 when not using any normalization.

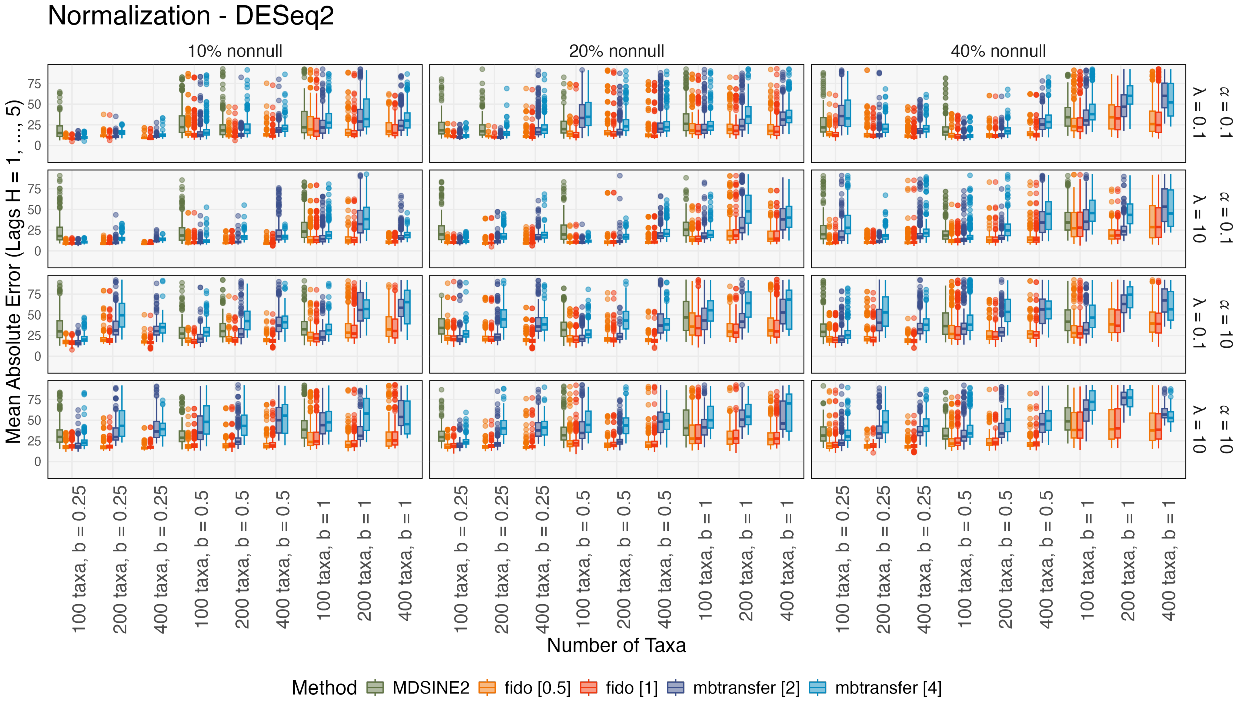

Fig F: The analog of Fig 4 when using DEseq2 size-factor normalization.

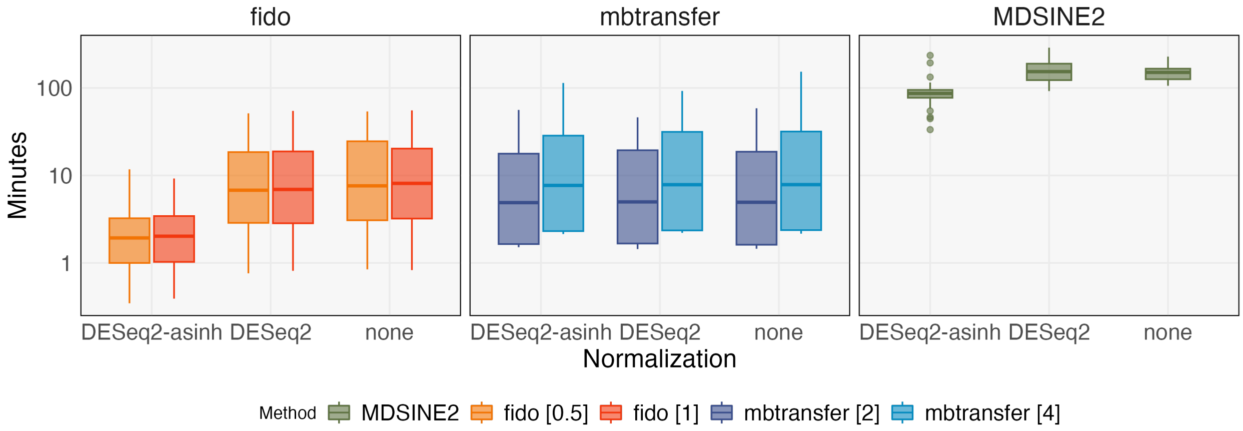

Fig G: Computation times for methods considered in the simulation experiment. fido is fast on untransformed, count data, which is the context in which it was originally designed. mbtransfer is comparable to fido on transformed data. Both packages are an order of magnitude faster than MDSINE2.

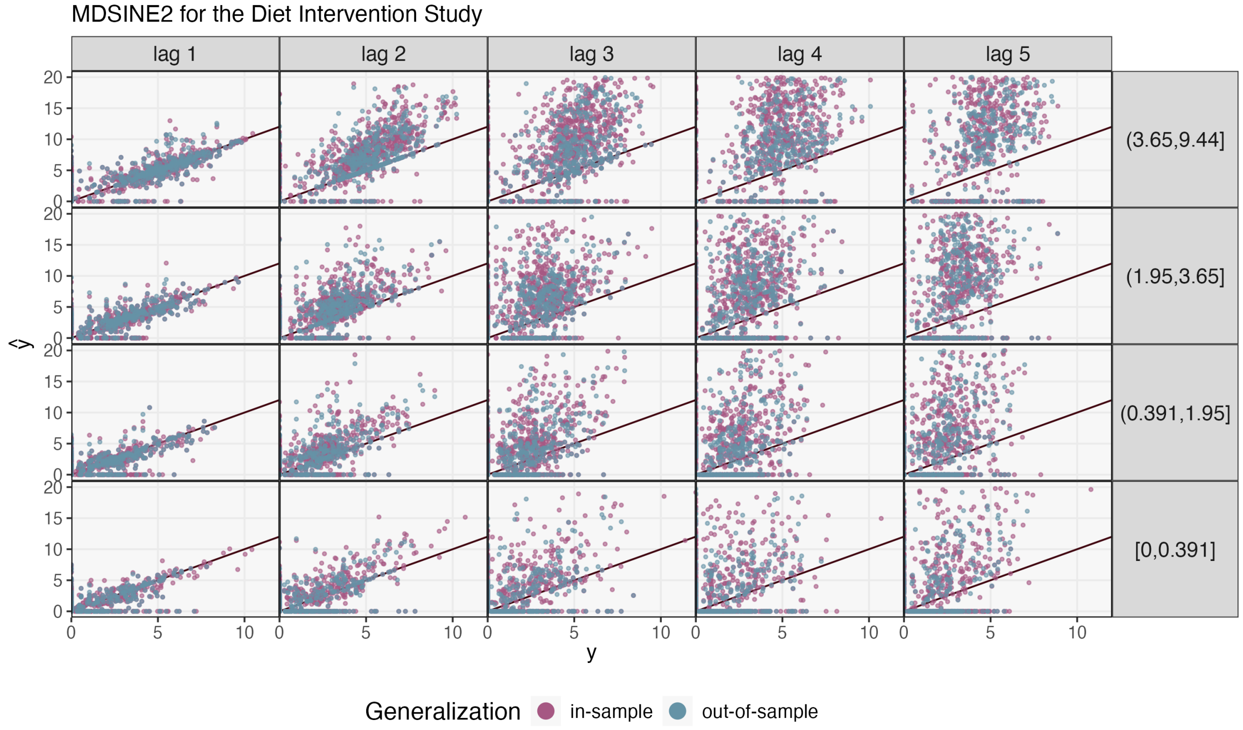

Fig H: The analog of Fig 7 when using MDSINE2. MDSINE2 is effective at short time horizons but can deteriorate over longer ranges. See also Table B for the correlations associated with each panel.

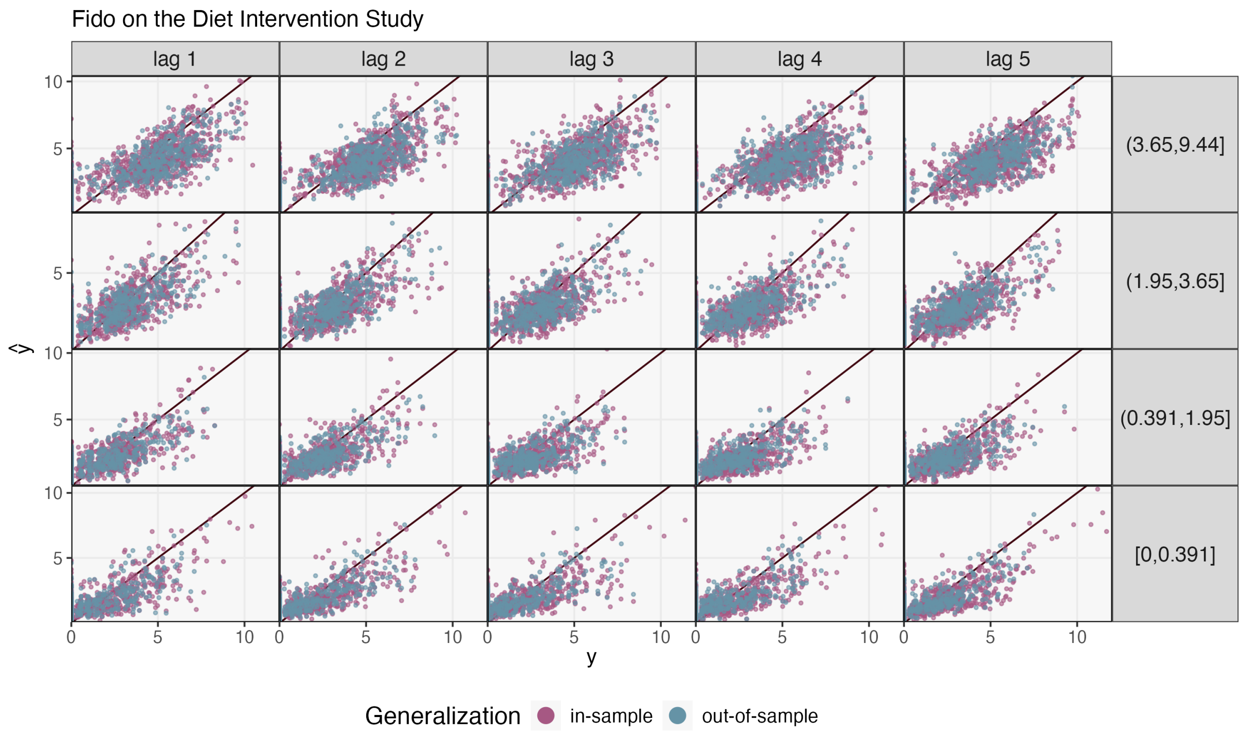

Fig I: The analog of Fig 7 when using fido. Performance on previously unobserved subjects is improved when using this approach, relative to either mbtransfer or MDSINE2. See also Table C for the correlations associated with each panel.

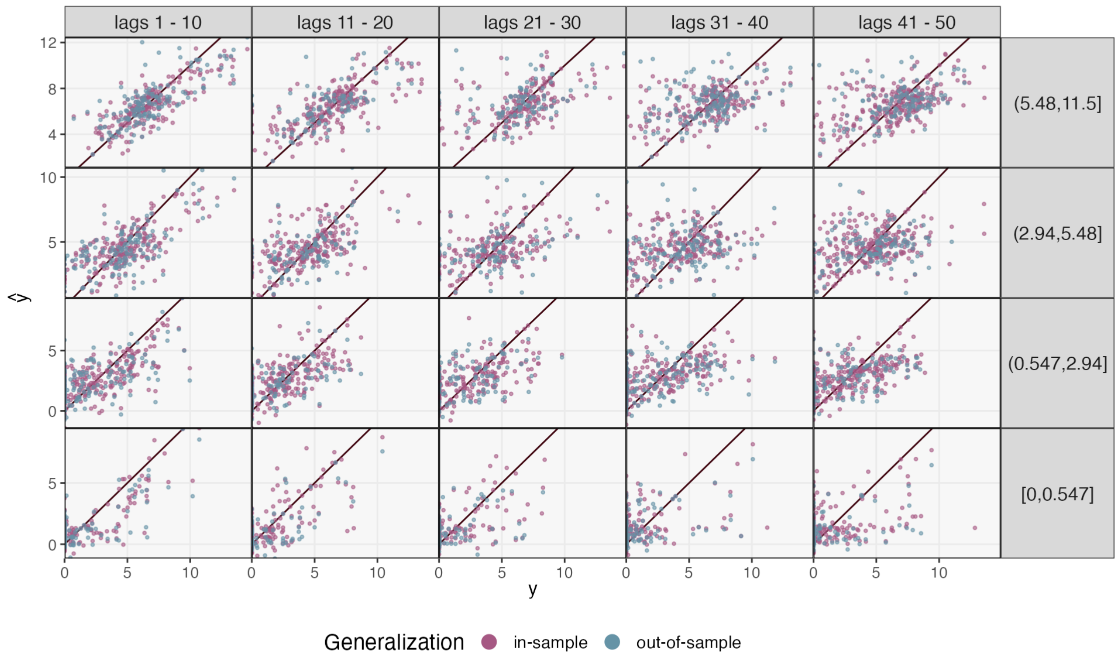

Fig J: The analog of Fig 7 when using mbtransfer for the postpartum case study. Each point is one sample. sample error refers to errors from future timepoints of subjects observed in the training data. new subjects errors are those on previously unobserved subjects. As before, errors predictions are most accurate on nearby time lags and more abundant taxa.

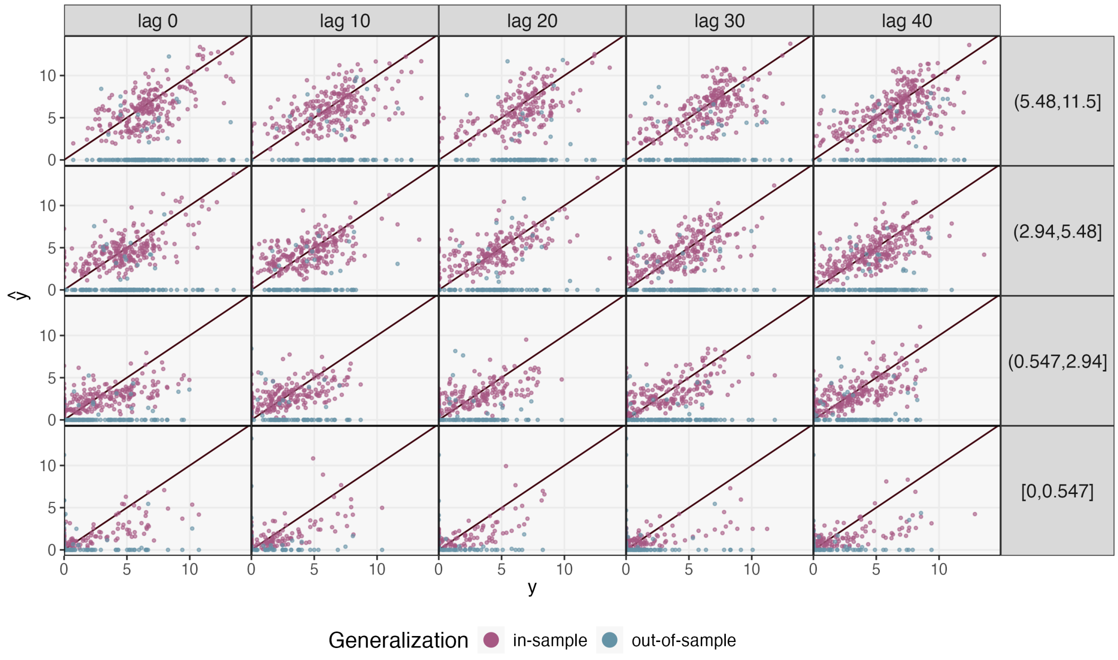

Fig K: The analog of S2 Fig J when using fido on the postpartum dataset. Note that some out-of-subject forecasts yielded missing values, and that these were imputed using the means associated with each taxon.

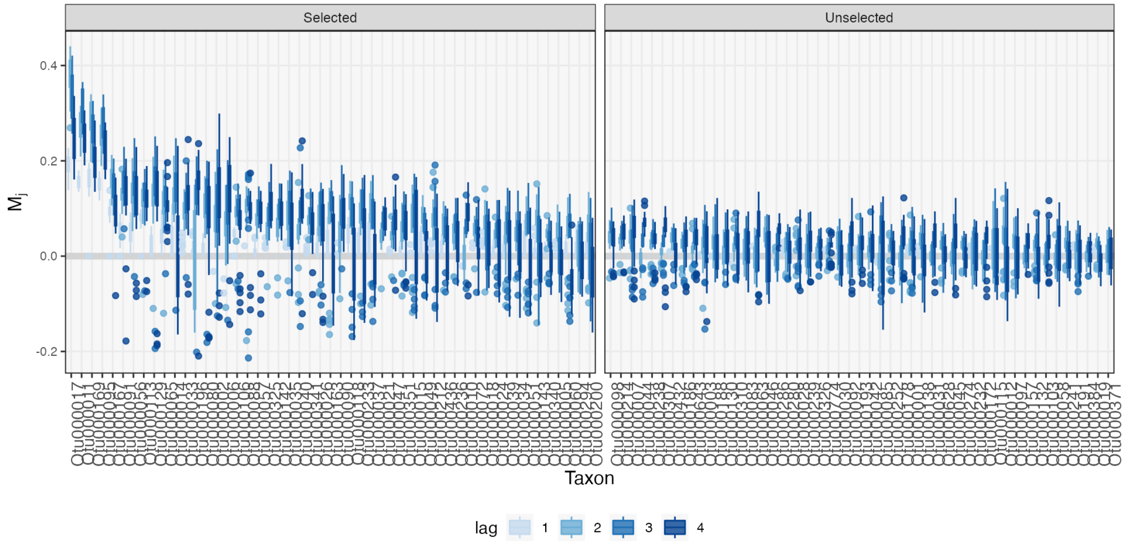

Fig L: The distribution of mirror statistics M_j for all selected and a subset of unselected taxa in the diet study. Larger statistics indicate strong, consistent lag-0 effects (specifically, $PD_j(0)$ for taxon j) across data splits. The selection threshold is chosen adaptively according to a false discovery proportion estimate. The unselected taxa shown are those with the largest median M_j, and we have shown as many as possible while limiting the total number of boxplots to 100.

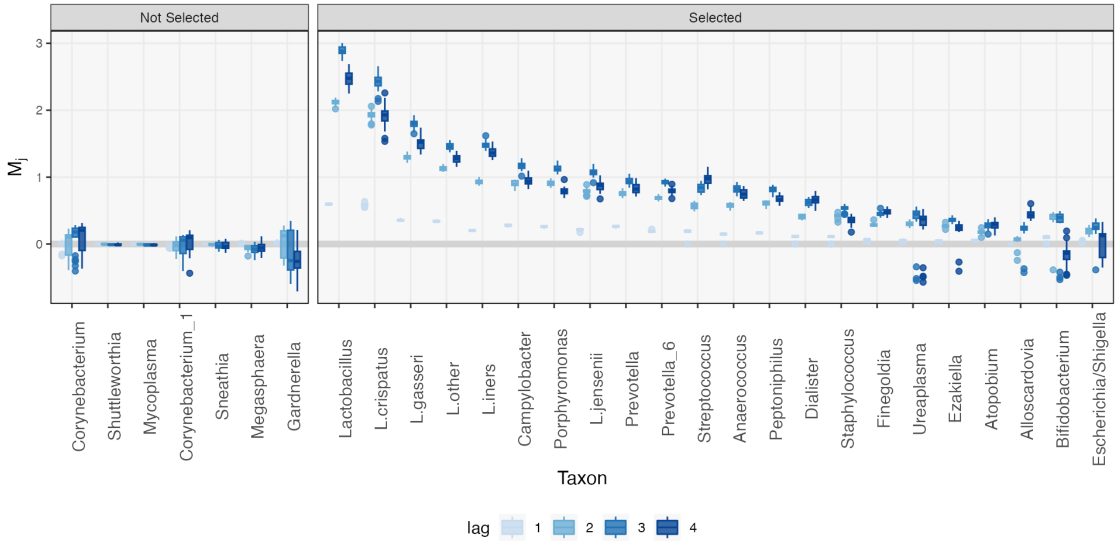

Fig M: The analog of S2 Fig L for the mirror statistics in the postpartum case study. Mirror statistics appear more concentrated, which is likely a consequence of the larger sample size and stronger effects visible in this dataset.

References

[1] Elizabeth K. Costello, Daniel B. DiGiulio, Anna Robaczewska, Laura Symul, Ronald J. Wong, Gary M. Shaw, David K. Stevenson, Susan P. Holmes, Douglas S. Kwon, and David A. Relman. Longitudinal dynamics of the human vaginal ecosystem across the reproductive cycle. *bioRxiv*, 2022.

[2] Lawrence A. David, Corinne F. Maurice, Rachel N. Carmody, David B. Gootenberg, Julie E. Button, Benjamin E. Wolfe, Alisha V. Ling, A. Sloan Devlin, Yug Varma, Michael A. Fischbach, Sudha B. Biddinger, Rachel J. Dutton, and Peter J. Turnbaugh. Diet rapidly and reproducibly alters the human gut microbiome. *Nature*, 505:559 – 563, 2013.

[3] Daii Yajima, Hiroaki Fujita, Ibuki Hayashi, Genta Shima, Kenta Suzuki, and Hirokazu Toju. Core species and interactions prominent in fish-associated microbiome dynamics. *Microbiome*, 11, 2022.
