# Supplementary material for: mbtransfer: Microbiome intervention analysis using transfer functions and mirror statistics
Source: PLoS Comput Biol. 2024 Jun 14;20(6):e1012196. doi: 10.1371/journal.pcbi.1012196 (PMC11210883; doi:10.1371/journal.pcbi.1012196)
Supplement: Attachment — Submitted filename: Response_to_Reviewers.pdf [file pcbi.1012196.s004.pdf]

Reviewer	Comment	Actions Taken
2	Page 4: “we extract non-overlapping taxonomic and intervention histories”. What does “non-overlapping” mean here?	Thank you for pointing out the ambiguity here. For estimation, we divide the sample trajectories into separate non-overlapping time windows. This ensures that there is no information leakage between the training and test sets. The taxonomic and intervention time segments are exactly matched. We have modified this language to: "For estimation, we tile sample trajectories into disjoint time windows. For each window, we extract aligned taxonomic $*Y_{t - 1}^{(i)}$ and intervention $*W_{t}^{(i)}$ samples."
2	Page 11: “the out-of-sample forecasts showed a clear correlation with the truth”. It would be more rigorous to show the statistics here.	The main text now includes the minimum and maximum correlations across these panels. We have also included correlations associated for each panel, case study, and method in Supplementary Tables 1 - 5. We have updated our package's vignettes to show how these tables were calculated for our method, and the paper's accompanying repository includes the table calculations for the baselines.
2	Page 11: “Across taxa, we found that the first and third quartiles of the counterfactual differences tended to agree”. It is unclear to me what the authors mean here and why it suggests that “the model did not detect interactions between the intervention effects and microbial composition”.	We apologize for the lack of precision. The strength of the intervention effect is comparable between the two birth control groups -- the word "interaction" in this context refers to the linear regression meaning of a set of covariates modulating one another's effects. We have clarified this and also explained which parts of the Figure 8 lead to this conclusion:<br><br>"Across taxa in Fig 8a, the ribbons for the birth control and no birth control groups generally overlap. Since these ribbons reflect the range of the estimated counterfactual difference across those groups, this implies that the model did not detect a relationship between the intervention's effect on microbial composition and birth control status. Therefore, the effect of the birth event may be uniform across baseline community profiles."
2	For mirror statistics, is that possible to compare it with a simple baseline statistics testing the abundance before and after perturbation?	Based on your suggestion, we have added a baseline that applies t-tests to the pre and post windows surrounding each intervention. The results for this approach are presented in the "Pre/Post t-test" panels in Fig 6. While we agree that this is a natural approach, our results indicate that DESeq2 and Mirror Statistics offer better power and FDR control across the settings considered in our simulation study. One challenge with the pre/post testing approach is choosing an appropriate window size. Larger windows offer the benefit of larger sample sizes but at the cost of reduced sensitivity to strong, transient shifts. In an effort to balance this trade-off, we opted for a window size of 8 timepoints.
3	1. A more detailed description of f, the autoregressive transfer function model, could be included in the methods section. a. How does f account for microbe-microbe interaction? Alternatively, how can the estimated f be used to study microbial interactions?	A new paragraph, starting with "What relationships are expressible within this model class?" has been added to the section "Transfer function models." We explain how the input for each model f[j] is created and summarize the functional form of the final set of boosting models. This clarifies the specification of the model f and the design choices involved.<br><br>The question of how transfer functions can be used to understand microbe-microbe interactions is important, and we now briefly explain how partial dependence profiles can be used for this purpose ("The fitted $\hat{f}_{j}$ can be analyzed..."). The new "Toy example" section, especially the partial dependence profiles in S2 Fig 2, illustrates how such microbe-microbe interactions can be interpreted. We view this as a somewhat experimental feature, though, since we haven't systematically evaluated inference for microbe-microbe interactions in the same way as we have for perturbation-microbe interactions. We added a comment to our discussion ("The partial dependence profiles in S2...") identifying this evaluation as an important future direction.

3	2. Is the model able to account for oscillatory behaviors such as those seen in an unstable 2-state predator-prey model?	In the new "Toy Example" section's opening paragraph, we discuss how the mbtransfer model class can account for oscillatory dynamics. Essentially, mbtransfer learns a high-dimensional regularized linear model with interactions between predictors. This section describes how a predator-prey model exhibiting oscillatory dynamics can be rewritten in a form compatible with our approach.<br><br>To illustrate this, we provide a small simulated dataset example. Although the model fit is not perfect (see our next response), it does appear to capture the competitive dynamics between the predator and prey species.
3	3. Perhaps an example of the model being applied to a small toy dataset of 2 variables will better illustrate how the model works.	We appreciate this thoughtful suggestion. The new "Toy Example" section illustrates how our transfer function approach could be applied to a predator-prey model exhibiting oscillatory dynamics. We believe this simple example effectively highlights both the strengths and limitations of our method. On one hand, it demonstrates the ability to capture the existence and directionality of relationships between species. However, it can also dampen or underestimate the true interaction effects.<br><br>This aligns with our intuition about the model's behavior. While highly expressive, it requires more data than parametric models specifically designed with certain interaction structures in mind.
3	4. How is counterfactual difference calculated? Is it calculated from steady-state abundance profiles with and without interventions?	The counterfactual differences are estimated using the averaged difference of short term trajectories with and without the interventions. We do this because the steady-state abundance profiles are not available in closed form for our more complex transfer function model. This definition had been included in the section, "Mirror statistics," but we acknowledge that we gave the formula without any explanation. We have added some discussion to motivate the formula in the text immediately following it: "Intuitively, this approximates..."
3	S2 Fig. 2 (Normalization-None) appears to be based on counts data. Can mbtransfer be used on relative abundance (Total-Sum-Scaling. normalization) datasets?	Yes, mbtransfer can be applied to relative abundance data. We have added a clarifying note to the section on transfer function models: "Alternative normalizations can be applied before defining $*y_{t}^{(i)}$ (size factor, total sum scaling, centered log-ratio), and their influence is considered in Section \ref{sec:simulations}."<br><br>While we considered adding TSS to the list of normalizations/transformations evaluated in our simulation study, we ultimately decided to limit it to three methods. The size factor normalization used by DESeq2 has a similar motivation as TSS scaling but is less sensitive to large changes affecting only a subset of taxa. Identifying effective normalizations for microbiome data is an important problem on its own. Unfortunately, finding the optimal normalization strategy for transfer function modeling methods like ours is beyond this manuscript's scope. To acknowledge this, we added the following note to the discussion: "Our simulation study revealed that forecasting performance depends on normalization strategy, and identifying the optimal normalization for transfer function modeling is an open problem."
3	Are both Fig. 7b and 8b based on interpolated data, as described in line 254?	Yes, both Fig 7b and 8b are both on the interpolated data. We had mentioned this preprocessing for the diet data in the introduction to that case study, but have emphasized this point in the captions for both figures:<br><br>Fig 7b: "(b) Subject-level data from the same taxa, with each row representing a subject and each column a timepoint, potentially interpolated from the original, unevenly sampled measurements."<br>Fig 8b: "Note that these data have interpolated to the biweekly level to account for uneven sampling times."

3	In Fig. 7, are there ways to test/highlight subjects with microbiome trajectory that is similar to the counterfactual difference? For instance, for OTU000098, only Animals 10 and 5 appear to exhibit a similar abundance trend.	We agree that heterogeneity in the intervention effect is evident in Fig 7. Unfortunately, our implementation of mirror statistics cannot identify specific individuals whose responses most closely resemble the estimated counterfactual differences. While we can imagine a sensitivity/influence function analysis that could potentially highlight samples driving the estimated counterfactual trajectory, deriving and validating such a procedure seems non-trivial, and we were unable to explore this direction in our revision.
3	Given the heterogeneity of the response in the dietary dataset, is there a confidence measure for the counterfactual difference calculated in Fig. 7a?	We also recognize this heterogeneity in the intervention effects from our visualization, but unfortunately mirror statistics do not come with an associated confidence measure. Philosophically, mirror statistics are more like knockoffs than post-selection confidence intervals, both of which were originally designed to assess significance of selected variables in lasso regression. Knockoffs provide selections and a false discovery rate guarantee for the selected set, but no inferential guarantees for the individual coefficients. Similarly, mirror statistics allow FDR-controlling selection of a set of promising effects, but no estimates of feature-wise uncertainty.
3	How were the datasets from the case studies normalized?	We apologize for ommiting this point -- we used DESeq2-asinh normalization for both case studies (e.g., line 71 in https://go.wisc.edu/e99gj4) We have clarified this point in both of the case studies.
3	How does mbtransfer fare against the other software in the case studies?	We have added comparisons to fido and MDSINE2 in our case study section. More detailed performance summaries and figures are also shown in S2 Tables 2 - 5 and S2 Fig 8 - 11. MDSINE2 forecasts always diverged on the postpartum dataset, which is why that baseline is missing. As in the simulation experiments, fido and mbtransfer are generally comparable, with differences in performance related to the overall sample size (mbtransfer is relatively better in large datasets) and quantile of taxonomic abundance (fido is superior for rare taxa). We discuss these and other differences in forecasting performance in the main text ("For reference, we generated analogous forecasts..." and "We also evaluated forecasts..."), with the intent that they can help potential users understand in advance which methods might be more suitable.
1	FDR control via mirror statistics is only guaranteed when key assumptions are satisfied. As the authors state, one assumption is the fact that the distribution of mirror statistics is symmetric about zero under the null. Another assumption, which the authors do not state, is that the mirror statistics are weakly correlated (Assumption 2.2 in Dai et al.). I am concerned that, in many practical settings, both assumptions may be violated in the authors applications. As I expand upon below, the authors should provide a more thorough study and discussion of when their method works and when it does not. So long as the authors provide an accurate picture of the limitations of their method, regardless of the results, I think this manuscript will be useful to the field. That said, in its current form these potential limitations are not adequately explored.	We appreciate the sharp observation about the influence of dependent hypotheses, which we had not previously explored. First, we have revised the text to discuss the weak correlation assumption, in addition to symmetry: "we propose a mirror statistics approach \cite{Dai2020FalseDR}, which supports variable selection under minimal assumptions, symmetry under the null hypothesis and weak dependence across tests, whose plausibility is discussed below" "In contrast to the symmetry assumption, weak dependence is less likely to hold in practice..."<br><br>Second, we have modified our simulation's data generating mechanism to allow for phylogenetically dependent hypotheses, discussed in more detail below. See the covariance matrices in S2 Fig 3. As you anticipated, we do observe inflated false discovery rates under some normalization regimes, which is included in the simulation discussion: "Higher phylogenetic correlation ($\alpha = 0.1$) inflates the false discovery proportion..."<br><br>However, in the regime we recommend (DESeq2 size-factor normalization and sufficiently many taxa), FDR control still appears valid even under quite strong correlation. This robustness was surprising, and we have aimed to clearly communicate the dependence assumption so that users understand its importance when applying mirror statistics.

1	To address this comment, I encourage the authors to expand their discussion of these assumptions to the manuscript and expand their simulation studies in two ways to provide at least some minimal degree of empirical results as to the robustness of their proposed approach to violations in these assumptions. First, the authors could incorporate varying degrees of phylogenetic structure between the taxa (e.g., phylogenetically patterned correlations in errors or in other confounding effects that might be present under the null). I would encourage the authors to explore how FDR might change as a function of that phylogenetic correlation (e.g., we expect more phylogenetic correlations when analyses are preformed at the sequence variant level than at higher taxonomic/phylogenetic levels).	We appreciate the concrete suggestions and made an effort to implement them faithfully. First, we modified our data generating mechanism to support correlated errors \epsilon ~ N (0, \Sigma), rather than the originally assumed independent errors. The structure of \Sigma is designed so that taxa together on a hypothetical phylogenetic tree exhibit more correlated errors, as discussed starting from "The covariance Σ is designed to capture phylogenetic correlation..."<br><br>As noted above, we find that correlation can induce inflated FDR in certain regimes, and this is discussed in the simulation results: "Higher phylogenetic correlation ($\alpha = 0.1$) inflates the false discovery proportion for mirrors..."<br><br>Each figure referring to "alpha" is showing the impact of phylogenetic correlation correlation. Unfortunately, we could not explore more than two levels of phylogenetic correlation, very strong and very weak. Together with the changes to investigate variable sequencing depth, we already have 4 times more simulation configurations to consider compared to our original submission, which was already quite computationally intensive.<br><br>Nonetheless, the regimes we chose to represent are extreme cases, and we hope they effectively capture the qualitative influence of phylogenetic correlation on our proposal's inferential performance. While more exhaustive analyses would be ideal, we believe our current results provide meaningful insights into this important factor.
1	Second, the authors could, and likely should, expand their simulation to incorporate the arbitrary sequencing depth artifacts present in microbiome data. The counts y_i are not independent as the simulation suggests. I suspect that if the negative binomial was replaced with a multinomial (or the counts y from the negative binomial were resampled multivariate hypergeometric to an arbitrary depth) then the authors model will see elevated rates of false positives. These problems will likely become most noticeable when then intervention causes the total microbial load to shift (see McGovern et al., 2023).	We have modified our data generation mechanism to allow control for variability in sequencing depths. Specifically, we now simulate from NB(b * exp(theta), ...) where the term b is drawn from either a concentrated or a spread gamma distribution. We present the results of these runs in the updated forecasting (Fig 3) and inferential (Fig 5) results. For the intervential results, we saw no substantive change in FDR or power. To avoid cluttering the figure, we do not graphically encode the depth variability parameter \lambda, though all settings are present. However, we do include \lambda in the forecasting results (Fig 3), as sequencing depth appears to influence prediction performance. In general, the relative performance of mbtransfer is higher in regimes with more variable sequencing depth.
1	Regarding the symmetry assumption, it is less clear how the authors could explore model violations. At present the authors simply state they assume the mirror statistics are symmetric about zero under the null. Notably this seems to rest on an assumption that the error \(\epsilon\) are symmetric about zero. As it is currently written, I do not understand why such an assumption would make sense. For context, Dai et al., make a strong argument that, under a wide range of settings, this assumption is likely true for certain mirror statistics based on linear model or graphical model estimates. The gradient boosting methods proposed are non-trivial and I do not think that prior arguments by Dai can be applied here. This needs further discussion and study. Doesn't a number of panels in figure 6 suggest that this assumption may be violated in practice?	We agree that the symmetry assumption is challenging to verify in practice. As we now clarify in the revised "Transfer function models," our setting is not far from the setting of Dai et al. In particular, we are not using tree-based gradient boosting models, which could indeed yield quite different mirror statistic behavior. Instead, we used regularized linear gradient boosting models ("gblinear" in xgboost). These are high-dimensional linear models, with nonlinearity only arising from the interaction terms we explicitly provide as input. Therefore, we believe that it is not unreasonable to apply the methods of Dai et al. to our setting.<br><br>Regarding your comment on Fig 6, this shows the true and predicted values, y and \hat{y}, on real data. The symmetry assumption relates to the mirror statistics derived from differences in counterfactual forecasts, which cannot be directly seen here. Did you perhaps mean S2 Fig 6? This can be used to check the distribution of the mirror statistics themselves. We would argue that they appear consistent with the symmetry assumption, as most unselected taxa have mirror statistics roughly symmetric around zero, aligning with the necessary behavior under the null.

1	Fido is not designed to perform this task and, as the main author of that package (Justin Silverman), I would not recommend it be used in that way. In fact, in our original paper we explicitly state that this is a method for log-ratio estimation. In other words, fido is designed to estimate linear and non-linear trends in the relative abundances (or log-ratio abundances) of taxa and not their absolute abundance. It is only recently (Nixon et al. 2023, and McGovern et al. 2023) that we have started bridging the gap between estimation of log-ratios and absolute abundances. In short, when you give fido data with absolute abundance information (e.g., negative binomial data rather than multinomial data) fido basically just throws it away as its expecting the data has arbitrary scale. As a result, the authors should report how they are comparing estimates of log-ratios to their ground truth which is basically changes in absolute abundances.	Thank you for sharing your expertise with fido. We acknowledge that fido was not designed for this setting. We found it worth considering because there are relatively few methods available for intervention analysis. Also, in our study, fido shows competitive performance.<br><br>We have added comments to the simulation section to contextualize our use of fido: "We caution that fido was designed for relative abundance..."<br><br>Also, our revised discussion highlights the need for more work characterizing the impact of normalization on assessing interventions in dynamic data (and the possibility of avoiding it, in line with the references you share): "forecasting performance depends on normalization strategy, and identifying the optimal normalization for transfer function modeling or understanding whether it is possible to bypass it entirely..."
1	Are they using CLR coordinates for this comparison? Others do this but we (and others) have repeatedly expressed concern with this practice (Nixon et al. 2023, and McGovern et al. 2023, or just about any article by Pawlowsky-Glahn or Egozcue).	No, we did not use a CLR transform. We agree that this would lead to problematic behavior. The only normalizations used are those explicitly discussed in the paper: no normalization, DESeq2 size factor-based, and DESeq2 size factors-based with asinh transformation.
1	Other non-trivial features that should be reported include the choice of kernel in fido. I am going to guess the authors are just using an RBF kernel in the basset model in fido. This is a common choice but I expect that other kernels might have better performance (again fine to leave as is and just explain choices).	We have revised the simulation introduction to clarify the choice of kernel: "Specifically, we applied fido with an RBF kernel with hyperparameters...." We also noted that these are defaults and that performance could be optimized by more systematically searching hyperparameters.
1	Both MDSINE2 and fido are Bayesian models. The choice of prior is non-trivial and should be reported. Note the default priors in fido come with an explicit warning to users urging them to think more deeply about their problem and design appropriate priors that reflect biological knowledge.	We have clarified in our simulation introduction ("Both MDSINE2 and fido are Bayesian models...") and in our discussion of the results ("However, as noted above, we have avoided extensive hyperparameter tuning and tailoring of the prior...") that the performance of both methods could be improved by design of a prior that reflects true initial belief about the scientific problem. We have referred the reader to S1 for more details about the assumed data generative mechanism and the parameters that could be adjusted to better match their problem of interest.
1	For MDSINE2, the model was designed to also use qPCR measurements to bridge the gap between relative and absolute estimation. How are the authors then using MDSINE2 here without simulating qPCR data?	We apologize for not including this in the original text. Yes, we simulated qPCR data. We chose values that are close to the qPCR values from the examples in the MDSINE2's documentation. We understand that this is not ideal, but it was the only way we could include this in our comparison. We now discuss this issue in the revised simulation introduction: "Note that MDSINE2 was designed assuming availability of qPCR..."
1	Finally, as a smaller point, it seems wrong to say that a limitation of fido is its computational speed (page 3) when later (page 9) you say that fidowas comparable and even in once instance faster than mbtransfer.	We actually cannot find this comment in the proof of the submitted manuscript. In the original text, the only place where we comment on fido's speed is when we say that it can be faster than mbtransfer: " fido and mbtransfer had comparable computation times except when using DESeq2-asinh transformed data. In this setting, fido was faster, but mbtransfer was more accurate."
1	I think introducing mirror statistics to the field is important as I think there are multiple potential uses for them in the field. That said, the authors barely introduce them or explain their novelty. I think adding a paragraph somewhere providing a bit more background knowledge to the reader would likely improve the impact of the authors work.	Thank you for this suggestion, and we have added a short subsection at the start of the discussion on mirror statistics that summarizes the algorithm for the linear model setting. We also added a supplementary figure that visually explains the main steps of the algorithm. We hope that it clarifies the intuition behind this procedure and helps users understand why the assumptions matter. Finally, we appreciate your supportiveness, and also think that the mirror statistics approach has potential for inference and reproducibility in microbiome research more broadly. We hope that this summary helps introduce more researchers to the idea.
1	On page 3 the PDF seems to be malformed and the sentence starting with "Nevertheless, these methods" is oddly bolded on not on any particular line. Same with the word "profiles a few lines down".	We actually cannot find this issue in the proof of the submitted manuscript. We have checked our current revision carefully for formatting problems.

1	On line 67 you say the errors need to be symmetric but later you say they need to be symmetric and centered about zero. I think the "about zero" likely needs to be added on that line. As this is important and implies absence of systematic bias in the estimator.	Thank you for the noting this inconsistency. We have added "about zero" to both instances.
1	"timepoints that are at least max(P,Q) apart" I didn't understand what the authors are trying to say with this phrase (page 5).	We apologize for the confusing phrase. This is how we implemented it in the code -- the relevant point is that the temporal patches are nonoverlapping. We have rephrased this to: "and nonoverlapping segments beginning at times t"
