# Supplementary material for: mbtransfer: Microbiome intervention analysis using transfer functions and mirror statistics
Source: PLoS Comput Biol. 2024 Jun 14;20(6):e1012196. doi: 10.1371/journal.pcbi.1012196 (PMC11210883; doi:10.1371/journal.pcbi.1012196)
Supplement: Attachment — Submitted filename: Reviewer Comments.docx [file pcbi.1012196.s003.docx]

The paper introduces an innovative approach to investigate microbiome dynamics, addressing two primary objectives: 1) modeling and predicting microbiome trajectories with and without interventions, and 2) identifying species impacted by interventions through differential abundance analysis. To accomplish the first objective, the authors employ a linear-autoregressive transfer-function model trained using gradient boosting. For the second objective, they utilize an inference technique that partitions the dataset into two groups, assessing the partial dependence of each taxa's abundance on the intervention within each group to derive a test statistic for significance determination of differential abundance.

The methods are adequately described, offering sufficient detail to comprehend the procedures. The data simulation process is also explained with clarity, ensuring that the simulated dataset emulates the richness and sparsity observed in experimentally obtained temporal microbiome profiles. Comparisons with similar software are conducted (MDSINE2 and FIDO for task 1, DESeq2 for task2), elucidating differences in both prediction performance and computational efficiency. Furthermore, the authors validated their models using distinct gut and vaginal microbiome datasets, providing insightful analyses derived from their model. These evaluations substantiate the applicability of the proposed mbtransfer method to microbiome datasets.

Below are some questions and concerns I have that wasn't addressed sufficiently.

1. A more detailed description of f, the autoregressive transfer function model, could be included in the methods section.
   a. How does f account for microbe-microbe interaction? Alternatively, how can the estimated f be used to study microbial interactions?
2. Is the model able to account for oscillatory behaviors such as those seen in an unstable 2-state predator-prey model?
3. Perhaps an example of the model being applied to a small toy dataset of 2 variables will better illustrate how the model works.
4. How is counterfactual difference calculated? Is it calculated from steady-state abundance profiles with and without interventions?
5. S2 Fig. 2 (Normalization-None) appears to be based on counts data. Can mbtransfer be used on relative abundance (Total-Sum-Scaling. normalization) datasets?
6. Are both Fig. 7b and 8b based on interpolated data, as described in line 254?
7. In Fig. 7, are there ways to test/highlight subjects with microbiome trajectory that is similar to the counterfactual difference? For instance, for OTU000098, only Animals 10 and 5 appear to exhibit a similar abundance trend.
8. Given the heterogeneity of the response in the dietary dataset, is there a confidence measure for the counterfactual difference calculated in Fig. 7a?
9. How were the datasets from the case studies normalized?
10. How does mbtransfer fare against the other software in the case studies?
